# Do cortical gamma oscillations promote or suppress perception? An under-asked question with an over-assumed answer

**DOI:** 10.3389/fnhum.2013.00595

**Published:** 2013-09-20

**Authors:** William Sedley, Mark O. Cunningham

**Affiliations:** Institute of Neuroscience, Faculty of Medical Sciences, Newcastle University Medical SchoolNewcastle Upon Tyne, UK

**Keywords:** gamma oscillations, magnetoencephalography, electroencephalography, perception, inhibition, tinnitus, epilepsy

## Abstract

Cortical gamma oscillations occur alongside perceptual processes, and in proportion to perceptual salience. They have a number of properties that make them ideal candidates to explain perception, including incorporating synchronized discharges of neural assemblies, and their emergence over a fast timescale consistent with that of perception. These observations have led to widespread assumptions that gamma oscillations' role is to cause or facilitate conscious perception (i.e., a “positive” role). While the majority of the human literature on gamma oscillations is consistent with this interpretation, many or most of these studies could equally be interpreted as showing a suppressive or inhibitory (i.e., “negative”) role. For example, presenting a stimulus and recording a response of increased gamma oscillations would only suggest a role for gamma oscillations in the representation of that stimulus, and would not specify what that role were; if gamma oscillations were inhibitory, then they would become selectively activated in response to the stimulus they acted to inhibit. In this review, we consider two classes of gamma oscillations: “broadband” and “narrowband,” which have very different properties (and likely roles). We first discuss studies on gamma oscillations that are non-discriminatory, with respect to the role of gamma oscillations, followed by studies that specifically support specifically a positive or negative role. These include work on perception in healthy individuals, and in the pathological contexts of phantom perception and epilepsy. Reference is based as much as possible on magnetoencephalography (MEG) and electroencephalography (EEG) studies, but we also consider evidence from invasive recordings in humans and other animals. Attempts are made to reconcile findings within a common framework. We conclude with a summary of the pertinent questions that remain unanswered, and suggest how future studies might address these.

## Introduction

### Background

The term “gamma oscillations” refers to periodic fluctuations, in the local field potential of a neuronal structure, at a rate of over 30–40 Hz (the exact lower limit varying between different reports). Definitions of upper frequency limits to the gamma frequency range are highly variable, ranging anywhere from 48 (Fujioka et al., 2009) to 300 Hz (Steinschneider et al., 2008). At a neuronal circuit level, multiple mechanisms have been demonstrated to underlie gamma oscillations; all of these are driven by a process of synchronized periodic inhibition generated either by inhibitory GABAergic interneurons or, in a more physiological context, their interactions with excitatory glutamatergic neurons (Whittington et al., 1995, 2011). Inhibitory functional roles of gamma oscillations could include an intrinsic “brake” to prevent excessive neural responses to intrinsic or extrinsic stimulation (Kirschfeld, 1992) and/or a mechanism to suppress behaviorally irrelevant stimuli or stimulus features. While the immediate action of GABAergic interneurons is clearly inhibitory, the effect of ensuing gamma oscillations on the neural systems in which they occur need not be. For instance, firing of excitatory neurons preferentially occurs during a particular phase of gamma oscillations, corresponding to the period of minimal inhibition. Therefore, the summated excitatory post-synaptic potentials (EPSPs) generated by these neurons could cross the threshold for triggering action potentials in postsynaptic cells more readily under conditions of periodic inhibition than if they fired uniformly without being subject to an inhibitory influence (Tiesinga et al., 2004). Such a mechanism would increase overall neural activity in the postsynaptic neural population and impart the same gamma rhythm to that activity.

Recent years have seen a substantial and growing interest in gamma oscillations, particularly with regard to their role in higher cognitive processes such as perception (Gray et al., 1989; Lachaux et al., 2005; Gross et al., 2007; Griffiths et al., 2010), attention (Gruber et al., 1999; Sokolov et al., 2004; Bauer et al., 2006; Ray et al., 2008) and memory (Osipova et al., 2006; Weinberger et al., 2006). In almost all published experiments on gamma oscillations, they increase in magnitude (reflecting increased power, synchrony or both) in the presence of the stimulus or process under study. As well as this positive, and almost ubiquitous, association with a large range of higher level processes, gamma oscillations have a number of properties that make them an attractive candidate neural correlate of high-level processes. These include their occurrence on a timescale of tens of milliseconds, consistent with that on which perception occurs, and their synchrony between anatomically separate neural assemblies, which has been proposed as a solution to the “binding problem” of consciousness (Singer and Gray, 1995). However, these observations alone fall vastly short of proving a generative role for gamma oscillations in high-level processes. While such a “positive” role seems possible given the available evidence, it is also plausible that gamma oscillations could have a very different role with respect to these processes, and could actually inhibit rather than facilitate them. Such a suggestion may seem counter-intuitive; we have major unsolved questions in neuroscience, such as how the brain generates coherent complex perceptions out of distributed individual elements, and gamma oscillations seem to be the best fit solution out of known phenomena. However there are also many other processes, lacking full explanations, that could as plausibly be *mediated* by gamma oscillations, many of which are “negative”; these could include suppression of behaviorally irrelevant stimuli, uninformative stimulus features or noise, preventing excessive neural activity or otherwise serving as a “gating” mechanism. All of these need to occur on the same timescale as high level neural processes and involve the co-ordinated action of neural assemblies, and thus could be fulfilled by gamma oscillations.

As an example of the distinction between “positive” and “negative” roles for gamma oscillations, let us consider the situation where a stimulus activates the visual system, and consequently the visual cortex receives a large amount of incoming information. This includes signals representing various retinotopic locations in terms of luminance, color, motion and local contrasts in these features. A “positive” role for gamma oscillations in processing this information could include grouping together all of these features that represent the same visual object so that they could be processed as a coherent whole. Thus, an enhancement of the local gamma activity would lead to an increased tendency to process a visual scene as a smaller number of objects, each containing a larger number of features. Conversely, suppressing gamma oscillations would lead to the visual scene being perceived as a larger number of separate stimuli, each containing fewer features. Such changes could be demonstrated, for instance, using a paradigm that presented visual stimuli that were ambiguous in terms of how many distinct objects they represented, and asking subjects to state how many objects were present in each trial. A different “positive” role for gamma oscillations could be that they facilitate the forward-transfer of information through the cortical hierarchy. Thus, enhancement of gamma oscillations would lead to stronger or faster conscious perception or behavioral responses to stimuli. Gamma suppression would have the opposite effect. A “negative” role for gamma could include the opposite of this role; namely that gamma oscillations could act to prevent the forward-transfer of certain stimulus-related information. This could, for instance, constitute a mechanism for suppressing the representation of behaviorally-irrelevant stimuli or stimulus features. Similarly, gamma oscillations could act to cause a similar suppression of stimuli or stimulus features at the local level, by suppressing their neural representations. Such a mechanism would be important for mediating competition between stimuli or stimulus features. If this were the case, enhancement of gamma oscillations would lead to reduced onward transmission of stimulus information, with the perceptual consequences of either fewer stimuli or fewer stimulus features being perceived, or stimuli being perceived as less salient. Although perceived as less salient, it would be likely that the perceived stimulus features were selected as those with the highest discriminatory value or behavioral relevance. Suppression of gamma oscillations would have the opposite effect, with larger number of stimuli or stimulus features being more saliently perceived, with a likely tendency toward poorer perceptual discrimination due to an inability to filter out irrelevant information.

Besides “positive” or “negative” roles, gamma oscillations could potentially serve roles that are neutral or variable with respect to their net effect on perception/cognition, serve multiple roles or be epiphenomena of different neural process.

### Types of gamma oscillations

*In-vitro* studies have identified multiple alternative cellular mechanisms for the generation of cortical gamma oscillations, which are driven by different interneuron types and have *distinct* functional correlates (Whittington et al., 2011). Ideally we would be to be able to directly relate gamma oscillations observed with MEG and EEG to their underlying neuronal mechanisms, but such distinctions are generally not currently possible in most studies. Some attempts have been made to delineate specific gamma frequency bands, most notably distinguishing “gamma” from “high gamma” (Edwards et al., 2005; Canolty et al., 2006; Ray et al., 2008). The thresholds for separating these bands have been somewhat variable, but there is some evidence from auditory cortex recordings for differing behaviors of these two frequency bands within the same experiment (Edwards et al., 2005). However most studies have not gone as far as this in specifically commenting on disparities between different gamma bands, so in this review we will not focus on subtyping gamma according to frequency band alone. One crucial distinction between types of gamma oscillation that needs to be made is between narrowband and broadband gamma. Recent studies using invasive recordings in macaque visual cortex (Jia et al., 2011; Ray and Maunsell, 2011) have clearly identified two modes of gamma oscillation. These are illustrated in Figures 2C,D, along with examples from the human literature on likely equivalents of each mode of gamma oscillation (**G–I**). The first is a broadband gamma, *typically* starting at 30 Hz and extending upwards to at least 160 Hz (Jia et al., 2011; Ray and Maunsell, 2011), which is predominantly transient following stimulus onset, and correlates positively with multi-unit activity. This is exemplified in Figure 2D. The correlation of broadband high-frequency gamma oscillations with multi-unit spiking activity is so strong that it has been proposed that they simply reflect spectral leakage from multi-unit activity (Jacobs et al., 2010). Recent recordings from rat hippocampus *in vivo* have found two types of local field potential fluctuations in the range of higher gamma frequencies, one being leakage from multi-unit activity and the other being a true oscillation (Scheffer-Teixeira et al., 2013); these types of activity had subtly different properties that made them distinguishable. The principal differences were that “true oscillations” occupied a distinct frequency band (albeit a broad one), rather than an indefinite frequency range, and occurred during a different part of the theta phase cycle. However, the information required to make this distinction is not available in most existing studies of gamma oscillations, and therefore in this review we do not attempt a separation of these types of broadband gamma-range activity. The second type of oscillation in the gamma range is a narrowband (or “bump”) gamma, centered around 40–50 Hz with a bandwidth of around 10–20 Hz, as illustrated in Figure 2C. This occurs only in response to certain stimuli, whose characteristics include relatively large size and strong luminance contrasts at particular spatial frequencies, one example of which is shown in Figure 2A. The magnitude of this type of gamma oscillation varies inversely with multi-unit activity (Jia et al., 2011; Ray and Maunsell, 2011), as illustrated in Figure 2F. Human MEG and EEG studies using similarly large visual stimuli with high luminance contrasts (e.g., gratings and checkerboards) elicit a type of gamma oscillation with similar characteristics (i.e., narrowband and persistent for the duration of the stimulus) that have a slightly higher center frequency than in the macaque of around 60 Hz (Adjamian et al., 2004; Muthukumaraswamy et al., 2009; Scheeringa et al., 2011). An example of such narrowband gamma oscillations in humans is shown in Figure 2G. Another feature of narrowband visual gamma is that it is unexpectedly strong, often representing the dominant change in the power spectrum over and above changes in lower frequencies which are usually orders of magnitude stronger (Hoogenboom et al., 2006). Studies in other sensory domains have not identified narrowband gamma oscillations as in the visual system. Auditory stimulus-induced gamma oscillations mainly occur at higher frequencies of above around 80 Hz, occupy a broader frequency range and generally occur predominantly transiently to stimulus transitions (Edwards et al., 2005; Griffiths et al., 2010; Sedley et al., 2012). An example of auditory cortex gamma oscillations in response to stimulus onset and a stimulus transition is shown in Figure 2I. While lower frequency gamma oscillations are sometimes detected in response to auditory stimuli they are less abundant, not occurring in electrocorticography (ECoG) studies in the absence of higher frequency gamma (Edwards et al., 2005; Griffiths et al., 2010), and in MEG studies requiring very large numbers of trials to detect (Fujioka et al., 2009). In auditory cortex *in vitro*, however, two anatomically and functionally distinct gamma generators operate (Ainsworth et al., 2011), one generating a 30–45 Hz rhythm and one a 50–80 Hz rhythm, but it remains to be seen how these relate *to macroscopically-recorded* stimulus-induced rhythms. Gamma in somatosensory cortex, *exemplified in* Figures 4D,E appears similar to that in auditory cortex (Bauer et al., 2006; Gross et al., 2007; Ray et al., 2008), being predominantly relatively high frequency, broadband and transient for hundreds of milliseconds following stimulus onset.

While it is not certain that the broadband mode of visual gamma oscillation represents the same underlying neural process as auditory and somatosensory gamma oscillations, there is no clear evidence that it represents a different process either. Therefore, for the purposes of this review, the only distinction we will make between types of cortical gamma oscillation is between “narrowband” gamma oscillations (in normal perception reported only in visual cortex in response to specific stimulus properties) and “broadband” gamma, representing all other types. We do not mean to imply that what we call “broadband” is either homogenous in its frequency spectrum or extends through the whole gamma frequency range, but just that it is broadband compared to the specific “narrowband” visual gamma and does not share its properties of persistence or sole association with specific stimulus properties. Gamma oscillations are usually quantified using a type of time-frequency transformation of either directly-recorded or reconstructed source data. Such methods commonly include wavelet analyses, and the multi-taper method fast Fourier transform (MTMFFT). In any time-frequency decomposition, a trade-off must be made between resolution in time and resolution in frequency, and this is determined by the parameters used for the analysis. It is therefore possible, in certain instances, to make oscillatory activity appear to be broader or narrow in its frequency band than it actually is. In conducting this review, we have therefore taken the apparent time-frequency parameters into account when categorizing reported gamma activity as broadband or narrowband. We have also taken into account the time course of gamma activity and the stimuli used to induce it. In most instances there has been concordance between these factors, and we have therefore been confident in attributing a “broadband” or “narrowband” label. Furthermore, many studies clearly measured both broadband and narrowband components, which were readily distinguishable from each other. In any unusual cases where it was not clear, we have stated that we cannot be sure which type of gamma oscillation is represented.

### Measuring gamma oscillations

Gamma oscillations are easily measured using invasive recordings, to the point that they can be used for functional mapping purposes akin to traditional robust responses such as event-related potentials (ERPs; Jerbi et al., 2009; Nourski et al., 2012). Non-invasive EEG and MEG can be used to detect equivalent patterns of gamma oscillations as recorded invasively, though with a vastly lower signal to noise ratio which often means that source space reconstructions are required in order to detect these gamma oscillations above noise (Dalal et al., 2008; Sedley et al., 2012). Several human MEG studies have found gamma responses with equivalent stimulus-dependencies to those found with invasive recordings in macaques, suggesting that these two different approaches are measuring the same underlying neural processes (Swettenham et al., 2009; van Pelt and Fries, 2013; Perry et al., 2013). A significant concern relates to the detection of visually-induced gamma oscillations using scalp EEG (Yuval-Greenberg et al., 2008). The study in question found that, in EEG with channels referenced to the nose or average reference (as were common practice), the appearance of transient broadband gamma oscillations could be generated in occipital electrodes around 300 ms after stimulus onset; these apparent “gamma oscillations” could be attributed entirely to ocular micro-saccades contaminating the EEG reference. Due to these serious concerns with the validity of EEG studies on transient broadband visual gamma using such methods, we will not include in this review EEG studies likely to be compromised by this issue. MEG studies, source space modeling studies and work on other sensory modalities should not be affected by these artefacts.

### Scope of review

**Figure 1:**
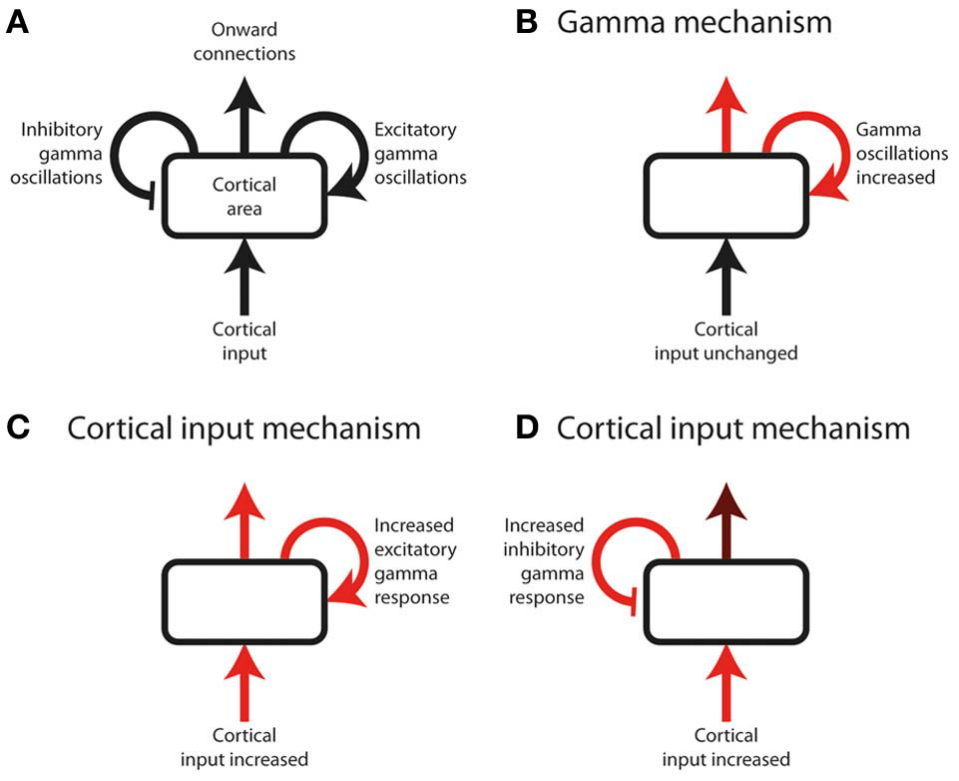
**Schematic of a hypothetical experiment, measuring cortical gamma oscillations, that risks being non-discriminatory. (A)** Annotated schematic of diagram format used in this figure. A cortical area (rounded rectangle) receives an input and sends out onward connections. Onward connections are influenced both by the cortical input, and by the effect of local gamma oscillations (curved arrows), which can either be excitatory or inhibitory with respect to onward connection strength. Onward connection strength can include perceptual or cognitive judgments, and therefore be inferred from behavioral responses as well as by measurement of downstream neural responses. Note that cortical input can represent bottom-up input from subcortical or lower cortical areas, or top-down input from higher cortical areas. **(B–D)** Three potential underlying sequences of neuronal responses in a hypothetical experiment. Red arrows indicate neural processes increasing in magnitude as a function of the experimental effect under study (e.g., response to a stimulus, or effect of selective attention). Gamma oscillations increase equally in all three examples, and therefore the magnitude or direction of gamma power change in isolation cannot be used to distinguish between the underlying neural systems; inferring the role of gamma requires knowledge of how the cortical input and output change, in addition to how gamma changes, as a function of the effect under study. **(B)** The cortical input is unchanged, but the effect under study is primarily mediated by an increase in gamma oscillations, which lead to increased onward connections. **(C,D)** The effect under study is primarily mediated by increased cortical input. Gamma oscillations increase as a mechanistic consequence of this increased input, irrespective of whether their role is excitatory or inhibitory. Determining the role of gamma therefore requires knowledge of the onward connection strength, which is more strongly increased (relative to the cortical input) if gamma is excitatory **(C)** than if it is inhibitory **(D)**.

Establishing the role of cortical gamma oscillations is important for understanding brain function, but also of practical importance, as abnormalities of gamma oscillations are present in pathological conditions such as phantom perception, epilepsy and schizophrenia. In these conditions, detection of abnormal gamma behavior could potentially help in diagnosis or subtyping, and correction of it could be beneficial therapeutically. For these purposes, gamma oscillations need to be detectable non-invasively, either with electroencephalography (EEG) or magnetoencephalography (MEG). This review presents a summary of selected experimental evidence on gamma oscillations occurring in cortex. Some examples are given of studies that do not help to distinguish positive from negative roles of gamma oscillations (“non-discriminatory” studies). The design of an archetypal non-discriminatory study **is** that a stimulus is presented and neural activity is compared between the stimulus and pre-stimulus periods. In such studies it would be clear that the neural responses could indicate *any* direct or indirect consequence of the stimulus. We highlight studies that are less obviously non-discriminatory, but share the confounding factor that input strength to the cortical area under study is likely to have increased as a function of the cognitive effect under study; thus observed gamma oscillations could reflect any downstream consequence of this increased input (see Figure 1). Subsequently, a discussion is presented of studies whose findings favor either a positive or negative role of gamma oscillations. We discuss gamma oscillations with respect to normal perception, and in the pathological contexts of phantom perception (tinnitus and phantom pain) and epilepsy. Cited evidence is kept as focused on human non-invasive imaging as possible but, where it is helpful in the interpretation of non-invasive imaging studies, some evidence is drawn from invasive recording studies in humans and animals. The convention in this review is that “increases” or “decreases” in “gamma” refer to changes in the amplitude or power of gamma oscillations, as opposed to changes in frequency or phase. In some instances we do refer to gamma frequency (i.e., frequency of the dominant spectral peak), and in these cases this is clearly stated.

## Normal perception

### Narrowband visual gamma

#### Non-discriminatory studies on narrowband visual gamma

Visual cortex narrowband gamma oscillations are a highly-studied phenomenon that occurs in response to visual stimuli with certain properties. These include large size, (Jia et al., 2011; Ray and Maunsell, 2011) high luminance contrast (with color contrast alone not eliciting any such gamma oscillations despite producing an equal magnitude blood oxygen level dependent [BOLD] response as measured with functional magnetic resonance imaging [fMRI]; Adjamian et al., 2008; Swettenham et al., 2013) and regularly-repeating luminance contrasts within a specific range of spatial frequencies (Adjamian et al., 2004). As this type of gamma is specific to a narrow range of stimuli, it is highly unlikely to represent the definitive neural correlate of a widely abundant process such as conscious perception. It is noteworthy that the stimulus conditions required to produce narrowband visual gamma are the same ones that induce visual illusions (such as of color and/or movement) and often lead to unpleasant subjective sensations (Adjamian et al., 2004). While it is likely that this type of gamma has a role with respect to such illusions, the association alone does not suggest in favor of either a causal or inhibitory role. Invasive recordings in primate visual cortex have found that the phase of narrowband gamma in V1 correlates with multi-unit spiking patterns in V1, and also both gamma phase and spiking patterns in V2 (Jia et al., 2013). Furthermore, V2 spiking was predicted much more by V1 than V2 gamma phase. Similarly, it has recently been shown that endogenous and stimulus-driven fluctuations in the frequency of narrowband visual gamma in macaque V1 are instantaneously mirrored by the frequency of gamma in V2 (Roberts et al., 2013). These results suggest a process of gamma-mediated gating of feed-forward activity, but in isolation are non-discriminatory about what the role of this process in perception. As well as local connectivity, narrowband visual gamma also exhibits long-range synchrony in the visual pathway under conditions of attention (Gregoriou et al., 2009). Human studies have found that the center frequency of narrowband visual gamma depends upon the local concentration of GABA (Muthukumaraswamy et al., 2009), is inversely correlated to the stimulus-induced BOLD response fMRI (Muthukumaraswamy et al., 2009) and is positively correlated to performance on visual orientation discrimination tasks (Edden et al., 2009). Such findings point to a functional role of visual narrowband gamma in stimulus selection, but not specifically to the nature of that role. Similarly, it has been found that the magnitude of visual stimulus-induced narrowband gamma oscillations in middle occipital gyrus immediately before and after a change in that stimulus positively predict the speed with which that change is detected (Hoogenboom et al., 2010). This suggests a functional role of narrowband gamma in efficient visual processing, but does not point toward the specific nature of that role. In a positive role, a larger gamma amplitude could lead to a stronger sensory representation of the stimulus and therefore faster change detection. Conversely, in an inhibitory role, increased gamma amplitude could act to better attenuate irrelevant stimulus features, facilitating a more rapid identification of changes in relevant features. Also noteworthy is the finding that the peak frequency of narrowband visual gamma is strongly heritable (van Pelt et al., 2012).

It has been found that selective attention to a particular visual stimulus increases the narrow-band gamma response to that stimulus in macaque V4 (Fries et al., 2001). It is worth emphasizing that attentional modulation of a neural response does not necessarily imply a facilitative role of that response in attention or perception; in many cases augmentation by attention could simply reflect a consequence of increased bottom-up or top-down input to the cortical area under study (see Figure 1). In the visual system there is evidence for modulation of pre-cortical activity as a function of attention (O'Connor et al., 2002), and there is also the possibility of V4 responses simply reflecting downstream consequences of different processes in hierarchically lower visual cortical areas. In this particular macaque study attention-related increases in gamma power were accompanied by decreases in beta-band power. This response pattern reflects an exaggeration of the usual response pattern to visual contrast stimuli (Hoogenboom et al., 2006), and as such could reflect a predictable response to attentional effects earlier in the visual hierarchy. Human MEG work on narrowband visual gamma oscillations in V1 found that attention did not increase these narrowband oscillations, but did cause a broadband enhancement in gamma power in the same cortical area (Koelewijn et al., 2013).

#### Evidence for a “positive” role of narrowband visual gamma

In a behavioral paradigm, in macaques, involving reactions to a change in an attended stimulus in the presence of a spatially separate distractor, it has been found that peristimulus narrowband gamma oscillations in V4 have a strong predictive effect on reaction time (Womelsdorf et al., 2006); increased gamma in neurons representing the attended stimulus predicted fast responses, and increased gamma associated with the distractor predicted slow reaction times. This observation could suggest a positive role of narrowband gamma in generating representations of stimulus change, but an effect carried forward from earlier in the visual hierarchy cannot be confidently excluded. Recent work, also in macaque visual cortex, has studied the effect of selective attention toward one of two competing stimuli on gamma oscillations (Bosman et al., 2012); gamma oscillations in V1 associated with the attended stimulus showed a stronger correlation with, and causal influence over, gamma in V4 than those associated with the unattended stimulus. This finding is consistent with gamma mediating stimulus selection, as discussed in section Non-Discriminatory Studies on Narrowband Visual Gamma, but favors a positive role for gamma in this process, since it is the gamma associated with the attended stimulus that appears to most influence onward activity. A study using visual grating stimuli of low luminance contrast (close to subjects' thresholds for conscious perception) compared neural response patterns between stimuli that were perceived and those that were not (Wyart and Tallon-Baudry, 2008). No systematic differences between stimuli were present between these categories. It was found that narrowband gamma responses were stronger in response to perceived vs. non-perceived stimuli. Attention was controlled for, and did not influence these narrowband gamma results. Interestingly, the overall gamma response was fairly broad band, but the perception-related gamma enhancement only occurred in a narrow frequency band. Similarly, a study of a single hemianopia patient, who only sometimes perceived stimuli in their hemianopia visual field, found that perceived stimuli were associated with stronger narrowband gamma responses than non-perceived repetitions of the same stimuli (Schurger et al., 2006). However, in the latter study only, other cortical responses were not reported, so one cannot be completely confident that the gamma response differences were not simply part of an exaggerated overall response pattern.

#### Evidence for a “negative” role of narrowband visual gamma

In considering narrowband gamma oscillations as an inhibitory process, one must consider the limited range of stimulus conditions under which such oscillations occur. As previously mentioned, these include regular repeating strong luminance contrasts within a certain range of spatial frequencies, but not equally-salient color contrasts, or weak luminance contrasts. Thus, if narrowband visual gamma serves an inhibitory role, it appears that there is something unique about multiple strong luminance contrasts within a comparatively large stimulus that needs to be suppressed. Both high luminance contrast and large size of a stimulus have been shown to bias visual processing toward that stimulus (Proulx and Egeth, 2008), so it is probable that these features also trigger particularly strong neural responses compared to other visual features. With this in mind, is seems possible that narrowband visual gamma oscillations might act to balance the processing of a visual scene by reducing the excessively strong representation of specific visual feature combinations that would otherwise be over-represented.

**Figure 2:**
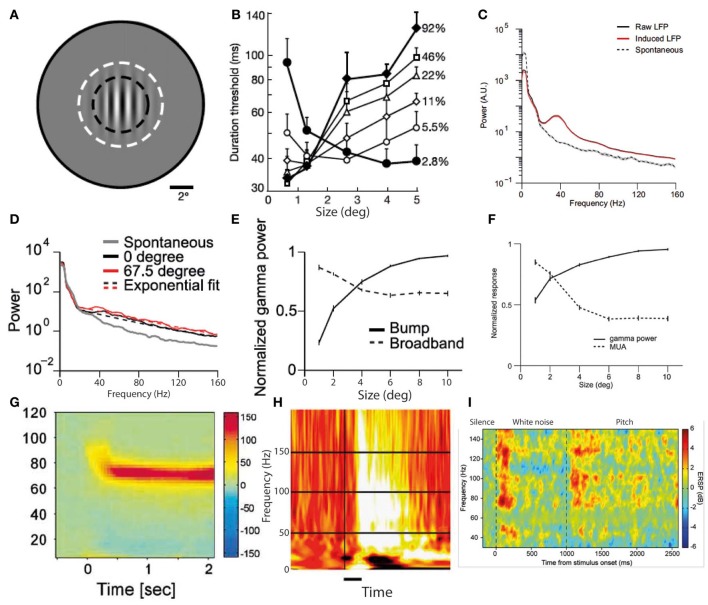
**Two predominant types of gamma oscillations: narrowband and broadband. (A)** Visual grating stimulus of type that produces both perceptual surround suppression and visual cortex narrowband gamma oscillations. **(B)** Duration thresholds, for detecting the direction in which the grating drifts, as a function of stimulus size (degrees) and stimulus contrast (%). Note that above 2° in size, thresholds become longer for high contrast stimuli, indicating surround suppression **(A,B** reproduced with copyright-holder's permission from Tadin et al., 2003). **(C)** Oscillatory power changes in macaque visual cortex in response to large visual grating stimuli (>2°), showing a prominent narrowband peak around 40 Hz, as well as a smaller broadband gamma increase. **(D)** Equivalent to **(C)**, but for smaller stimulus (<2°). Note that only a broadband gamma increase occurs. **(E)** Antagonistic relationship between narrowband (“bump”) and broadband gamma power in response to visual grating stimuli of increasing size. **(F)** Antagonistic relationship between narrowband gamma power and multi-unit activity in response to visual grating stimuli of increasing size (**C–F** reproduced with copyright-holder's permission from Jia et al., 2011). **(G)** Example of narrow-band gamma oscillations in visual cortex, recorded with MEG, in response to a visual grating stimulus (reproduced with copyright-holder's permission from Hoogenboom et al., 2006). **(H)** Example of broadband gamma oscillations in visual cortex, recorded with ECoG, in response to face stimuli (reproduced with copyright-holder's permission from Lachaux et al., 2005). **(I)** Example of broadband gamma oscillations in auditory cortex, recorded with depth electrodes, in response to a long stimulus with a transition from white noise to pitch at 1000 ms. Note the impersistence of gamma oscillations during the white noise segment despite a salient ongoing percept (reproduced with permission from Sedley et al., 2012; data originally published in Griffiths et al., 2010).

**Figure 3:**
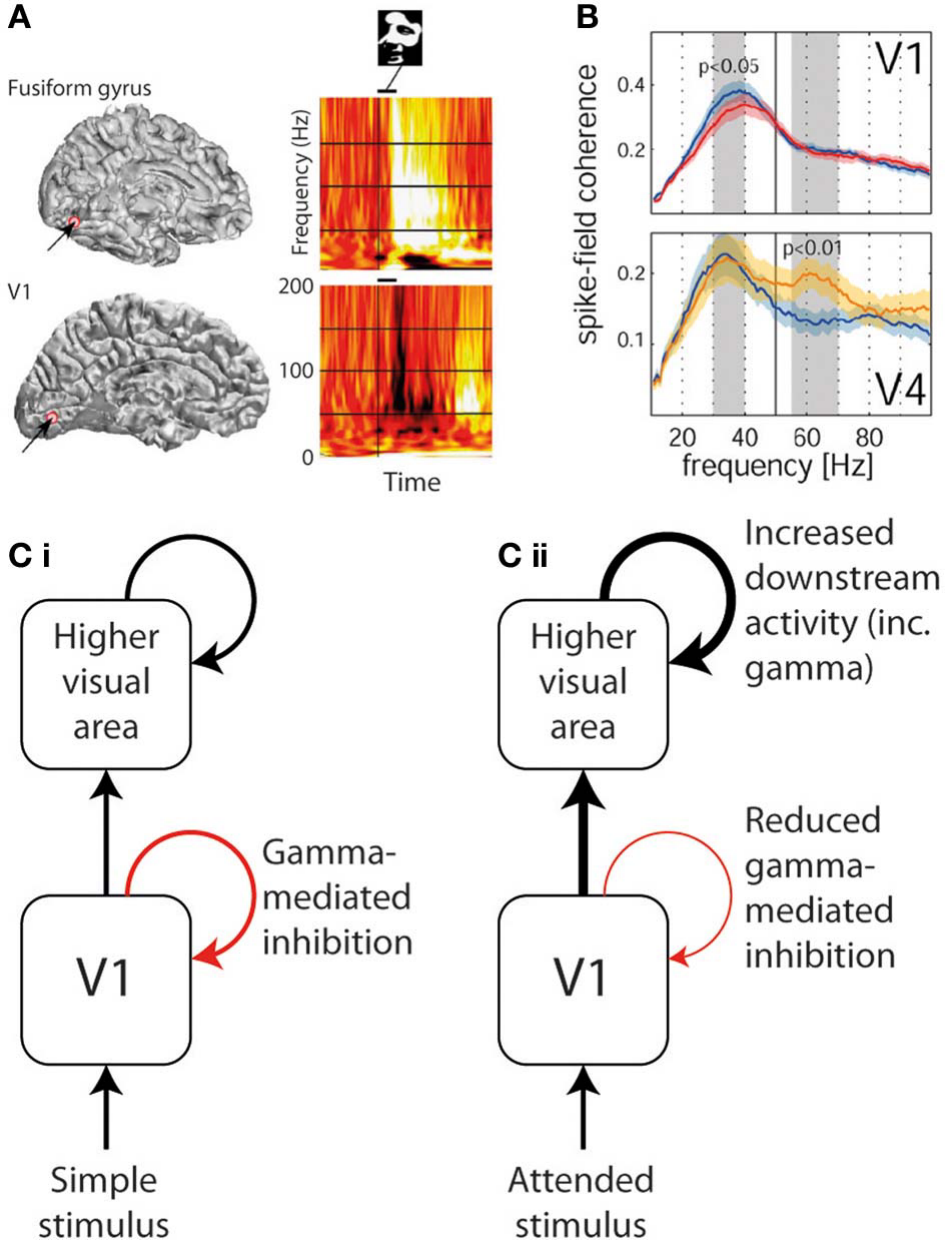
**Divergent trends in gamma oscillation responses in different areas of visual cortex. (A)** Gamma response to a face stimulus in V1 (lower—showing predominantly narrowband gamma power decrease) and in fusiform gyrus (upper—showing broadband gamma power increase) in the same human patient (reproduced with copyright-holder's permission from Lachaux et al., 2005). **(B)** The effect of selective attention (blue plot = unattended, red/yellow = attended) on gamma power in V1 (upper—showing narrowband gamma decrease) and in V4 (lower—showing gamma increase in a higher broader frequency band; reproduced with copyright-holder's permission from Chalk et al., 2010). **(C)** Schematic of how above findings can be reconciled if narrowband gamma oscillations are considered an inhibitory process. (i) In response to most basic passively-viewed stimuli, inhibitory narrowband gamma occurs in V1, but onward activity is still conveyed to higher visual areas also triggering gamma oscillations. (ii) In response to explicitly or implicitly attended stimuli, inhibitory narrowband gamma oscillations are reduced, leading to increased onward activity being conveyed to higher visual areas, which in turn triggers stronger gamma responses.

Although a direct experimental comparison has not been performed, it is noteworthy that the conditions required to generate narrowband visual gamma appear to be identical to those necessary to cause the perceptual phenomenon of surround suppression (Tadin et al., 2003): i.e., large size (above around 2°), high luminance contrast and particular spatial frequencies. This phenomenon involves poorer performance on perceptual discrimination tasks involving larger but otherwise equivalent stimuli (i.e., the larger stimuli contain all the information found in the smaller ones and more, without any conflicting features, yet are associated with worse performance). Supportive of such a role of narrowband visual gamma in mediating surround suppression is the finding that progressively increasing the size of a high-contrast visual stimulus into neurons' suppressive surrounds increases narrowband gamma while reducing multi-unit activity in macaque V1 (Gieselmann and Thiele, 2008). Figure 2 illustrates surround suppression, in terms of a *typical* causative stimulus **(A)**, the psychophysical effect **(B)**, and the antagonistic relationship between narrowband gamma and other measures of local neural activity **(E–F)**. It also bears mention that as well as narrowband gamma oscillations only being described in the visual system, perceptual surround suppression has likewise only been demonstrated in the visual system. Further to the demonstration of increased gamma in macaque V4 as a function of selective attention, similar experiments recording *simultaneously* from V1 found that selective attention was associated with *reduced* gamma oscillation spike-field coherence (SFC) in V1, yet still showed increased gamma in V4 as previously found (Chalk et al., 2010). These findings are illustrated in Figure 3B. This observation is incompatible with pre-cortical activity changes carried forward, pointing instead toward a role of gamma in inhibiting cortical responses and an effect of attention being a release from gamma-mediated inhibition. Such an explanation would propose that the release from inhibition in V1 would lead to increased input to V4, and therefore increased narrowband gamma in V4 as a downstream consequence of this (see Figure 3C). In perceptual terms, the attention-related disinhibition in V1 might increase the volume of stimulus-related information reaching V4, which would then be acted on by enhanced inhibitory responses that would inhibit irrelevant or excessive stimulus representations. Alternatively it could be that narrowband gamma serves different roles depending on the visual area in which it occurs. Consistent with these findings, human ECoG work using monochrome face stimuli found that early visual areas showed a decrease in gamma power coinciding with face presentation, while higher visual areas showed gamma power increases (Lachaux et al., 2005), as shown in Figure 3A. However, it is worth noting that the gamma frequency bands in this study were not definitely comparable to the previously mentioned macaque studies; early visual area power decreases appeared to include broadband and narrowband components, while increases in later visual areas were more broadband. Further support for a role of narrowband visual gamma in perceptual inhibition comes from recordings from cat V1; in a study of various interocular rivalry conditions, narrowband gamma responses to a visual stimulus increased dramatically where a second competing stimulus was added (Fries et al., 2002). The finding favors a role of gamma in mediating stimulus competition, possibly through inhibition of the “losing” stimulus, as opposed to a role in generating the percept of the “winning” stimulus (if this were the case the gamma change between conditions should be in the opposite direction or absent).

### Broadband gamma oscillations in normal perception

#### Non-discriminatory studies on broadband gamma oscillations

Broadband gamma power does not occur in isolation, but has been found, in multiple cortical areas, to be heavily influenced by the phase of low-frequency theta oscillations, preferentially occurring at a particular point in the theta cycle (Canolty et al., 2006). Such an observation is compatible with gamma being a response to cortical inputs (reflected as theta oscillations), but such an explanation is far from certain, and many other explanations exist. Broadband gamma has also been shown to be tightly coupled to multi-unit activity and the BOLD response (Mukamel et al., 2005), again highlighting its close relationship with other commonly-employed measures of neuronal population activity. Intracortical recordings in humans have identified prominent stimulus-induced gamma oscillations, in response to simple monochrome shapes, in lateral occipital (LO) and fusiform gyrus regions bilaterally (Tallon-Baudry et al., 2005). These gamma responses were generally of a broader frequency range than the narrowband gamma described in section Narrowband Visual Gamma, and only in some cases had any particular spectral peak, though notably did persist for the duration of the stimuli. The effect of selective attention had no unifying effect on gamma oscillations, in LO increasing baseline gamma power and reducing stimulus-induced gamma power, while in fusiform gyrus attention increased stimulus-induced gamma. Also of note were the different frequency ranges of gamma in the two regions within individual subjects during the same stimuli. The effect of attention on visual gamma oscillations has also been studied non-invasively; attention toward a rotating stimulus was found to increase occipito-parietal scalp EEG power by 10%, (Gruber et al., 1999) and selective attention toward either the auditory or visual modality increased gamma power in MEG sensor space corresponding to the relevant cortical area (Sokolov et al., 2004). As discussed previously, it is difficult to infer a particular role for gamma oscillations based on these findings; while they could indicate that gamma has a role in promoting attention or perception, they could also simply indicate a response to increased subcortical activity, or a response in non-primary sensory cortex to increased input from primary cortex. In auditory cortex, both brief externally-presented tones and the absence of expected tones lead to the generation of transient gamma oscillations detectable with MEG (Fujioka et al., 2009). Standard and deviant tone responses recorded with ECoG have found gamma responses, without showing distinct spectrotemporal profiles but with different spatial distributions (Edwards et al., 2005). These findings are fairly nonspecific with respect to the role of gamma, but do demonstrate a role over and above a mechanistic response to stimulus features. Responses to transitions from white noise to temporally-regular auditory stimuli, some of which elicited a percept of pitch, find that only pitch-producing stimuli elicited a significant gamma response (Griffiths et al., 2010), as illustrated in Figure 2I. This gamma response was strongly present following the onset of pitch, but was only sustained for the duration of the stimulus in cases where the stimulus was stochastic, in that its fine-grained pitch-containing information continually varied. The same study found that responses to white noise auditory stimuli elicited transient but not sustained gamma responses. Conversely, significant ERPs to the same stimuli were present to both pitch-producing and non-pitch stimuli. The same stimuli studied using MEG also find an equivalent response profile (Sedley et al., 2012). While these findings support a role for gamma in establishing perceptual pitch representations, they point against gamma being necessary for the maintenance of either the pitch percept or an auditory percept in general. A recent audiovisual cross-modal study on speech perception found that gamma oscillations were most strongly elicited by incongruence between the word currently being heard and the preceding movements of the speaker's mouth (Arnal et al., 2011). Such findings demonstrate a role in perceptual integration, but do not discriminate between the gamma oscillations representing an enhanced percept due to a surprizing stimulus, or an attempt to compensate for that surprizing stimulus.

#### Evidence for a “positive” role of broadband gamma

As mentioned previously, intracranial human recordings have found enhanced broadband gamma responses, as shown in Figure 2H, to visual stimuli that elicit a gestalt percept (impression of a meaningful coherent visual object as opposed to a collection of abstract features) compared to responses to non-gestalt stimuli that are otherwise equivalent (Lachaux et al., 2005). MEG recordings of visual cortex gamma during encoding of visual stimuli find that the magnitude of peristimulus gamma oscillations positively predicts the subsequent recall of these stimuli (Osipova et al., 2006). Human ECoG recordings to tactile and auditory stimuli find increased induced gamma oscillations in auditory or somatosensory cortex are associated with attention to that particular sensory modality (Ray et al., 2008). This gamma enhancement followed a different time course to any changes in ERPs, mainly occurring after the tail end of the main gamma response, and in a minority of cases gamma responses only being present at all during attention. These findings of a positive association between gamma magnitude, subsequent recall and attention imply that gamma could be involved in the attribution of behavioral or perceptual importance to sensory objects, however, in isolation they do not absolutely exclude increased inputs to the relevant cortical areas as a cause. Recent human ECoG work has helped to address this question by demonstrating that the attentional enhancement of induced gamma oscillations sequentially increases up the levels of the visual cortical hierarchy (Davidesco et al., 2013), suggesting an intrinsic cortical process rather than a secondary consequence of increased cortical input. Interestingly the latency of gamma enhancement was *shorter* in higher visual areas, implicating top-down as well as bottom-up processes. MEG evidence is supportive of a positive role for gamma in somatosensory attention. Somatosensory stimulation in general elicited a response pattern in primary somatosensory cortex of gamma enhancement, beta suppression and subsequent beta rebound; attention enhanced the gamma changes but reduced the beta changes (Bauer et al., 2006), suggesting a primary role for cortical gamma rather than a response to altered cortical input, which would have affected the gamma and beta responses equivalently. Other MEG work on somatosensory processing has focused on presentation of laser stimuli around the pain threshold; repetitions of the same stimuli perceived as painful elicited a significantly stronger gamma response than non-painful ones (Gross et al., 2007). ERP magnitudes were the same in both conditions, making cortical input an unlikely candidate to explain the gamma changes. MEG recordings in a visual paradigm found that selective attention to a stimulus was associated with increased broadband gamma oscillations but no change in narrowband gamma (Koelewijn et al., 2013), again suggesting a primary role of broadband gamma rather than consequences of altered cortical input. A human study, using ECoG and a backward-masking paradigm (which resulted in only some stimuli being recognized), found that when stimuli were recognized a rapid burst of gamma oscillations occurred in appropriate visual areas to the stimulus type, and this persisted for hundreds of milliseconds, well beyond the end of the stimulus (Fisch et al., 2009).

#### Evidence for a “negative” role of broadband gamma

There is limited human evidence for broadband gamma having a negative role. It has been shown that prestimulus gamma power in visual cortex, recorded intracranially, has a negative effect on the ERP elicited by the subsequent stimulus (Privman et al., 2011). While this suggests a forward-inhibition effect related to gamma, it does not rule out the possibility of gamma being excitatory, with increased baseline gamma therefore leaving cortex less responsive to external stimuli on account of already being relatively activated. Work in rats has used stimulation of the nucleus basalis to exert cholinergic control over auditory cortex gamma oscillations during sensory stimulation, with recall being tested in a subsequent session (Weinberger et al., 2006). The degree of gamma increase correlated with the subsequent specificity of recognition of the stimulus; i.e., animals showing greater gamma increases during learning tended to show recognition responses that were more specific to the learned stimulus, while those with smaller *gamma* increases showed *inappropriate* recognition responses to a wider range of stimuli beyond *just* the learned one. Such a finding is consistent with gamma oscillations having a role in preventing the formation of excessive and inappropriate memories, as in the experiment just described animals with the lowest gamma oscillations falsely “recognized” stimuli that were actually novel. While such a role would be important for the healthy functioning of perceptual and mnemonic systems, it would constitute a negative role on account of limiting downstream responses. Earlier work in the antennal lobe of insects had yielded similar findings; disruption of what were proposed to be an equivalent of mammalian gamma oscillations, along with sensory stimulation, resulted in reduced specificity of downstream neural responses, but normal responses at the level being disrupted (Macleod et al., 1998).

#### A unifying role for broadband gamma oscillations?

With broadband gamma oscillations being positively associated with such a wide range of perceptual and cognitive processes, it seems likely that their role is something that is relatively ubiquitous in terms of brain function. For reasons including the tight coupling with other measures of neural population activity, it has been proposed that broadband gamma represents nothing more specific than activation of a neural population (Merker, 2013). The evidence we have discussed is consistent with this theory, but by no means rules out alternative explanations. One account that is consistent with the findings we have discussed is the theory of predictive coding (Friston, 2005; Friston and Kiebel, 2009; Bastos et al., 2012). This general theory of brain function is distinct from *generative* models of brain function, which posit that neural structures continually generate representations of sensory objects. Instead, predictive coding proposes that the brain's perceptual centers act only to represent any discrepancies between the current state of the sensory environment and the brain's existing predictions about the state of the sensory environment. Such a system would involve massively reduced computational loads compared to generative sensory systems. Predictive coding accounts posit that the brain's perceptual systems operate in a hierarchical manner, with higher levels of the hierarchy generating predictions of the sensory environment, which are passed down to lower levels, and lower levels generating prediction errors (mismatches between predicted and observed activity patterns) which are passed up to higher levels. Discrepancies between predictions and incoming perceptual information therefore tend to be rapidly resolved by just one or two levels of the processing hierarchy. In such accounts, only changes in stimuli (or deviations from expectation) are associated with oscillatory power changes. This is because the neural representation of a persistent unchanging stimulus quickly reaches an equilibrium where predictions of the stimulus state are always accurate, and therefore neither prediction errors nor updated predictions need to be generated. More recent reviews on predictive coding have proposed that it is associated with specific oscillatory signatures, with beta oscillations representing downwardly-conveyed sensory predictions and gamma oscillations representing upwardly-conveyed prediction errors (Arnal and Giraud, 2012; Bastos et al., 2012). A predictive coding account of broadband gamma oscillations would expect gamma oscillations to occur during any stimulus transition, unless it were perfectly anticipated, during any deviation of stimulus features from what were expected, and possibly during conditions of increased attention (which could be achieved by increased weighting being given to prediction errors associated with the attended stimulus). These expected observations, based on a predictive coding model, are very much in keeping with observed experimental behavior of broadband sensory gamma oscillations; e.g., the transient nature of broadband gamma responses (Griffiths et al., 2010), the occurrence of gamma oscillations following the omission of missing stimuli (Fujioka et al., 2009) and the increased magnitude of gamma oscillations to incongruent as opposed to congruent audiovisual stimuli (Arnal et al., 2011). Unlike generative perceptual models, where gamma magnitude would be in proportion to stimulus salience, predictive coding accounts would expect gamma activity to be strongest in response to the most surprizing or unexpected stimuli. Theoretically the communication between beta and gamma frequency bands between cortical regions should be detectable using a model of effective connectivity such as dynamic causal modeling (Chen et al., 2008); the studies we have cited have not done this, but recent research has successfully modeled beta-gamma coupling between visual and motor cortex in imagery tasks (Van Wijk et al., 2013). Another attractive feature of predictive coding accounts of gamma oscillations is that, as they are argued to represent the magnitude of prediction error, they could be either positively or negatively correlated to perceptual salience, depending on the direction of the prediction error (i.e., predicting an erroneously weak or strong percept respectively). However, limited direct evidence exists to link gamma oscillations to predictive coding, so at present it remains just an attractive theory with respect to the role of broadband gamma.

## Phantom perception

### Introduction to phantom perception

Phantom perception can technically be considered a form of hallucination, in that it involves the perception of a sensory object that does not result from stimulation from the environment. However, in practical terms it can be typically be distinguished from more complex hallucinations on the basis of involving simple percepts, and resulting from neural changes following de-affarentation of sensory systems (Jastreboff, 1990; De Ridder et al., 2011a). This is compared to hallucinations in organic and psychiatric disorders that are complex and are thought to result in many cases from the misattribution of internally generated sensory representations (Friston, 2012; Nazimek et al., 2012). The phantom perceptual condition in which gamma has been most studied is tinnitus (persistent “ringing” in the ears), which tends to be initiated by deprivation of input to the auditory pathways; tinnitus patients with a “normal audiogram” have always shown deficits in more subtle tests of cochlear function when tested (Weisz et al., 2006; Schaette and McAlpine, 2011). In this review we largely focus on tinnitus, as this makes up the majority of the literature on gamma oscillations in phantom perception.

### Non-discriminatory studies of gamma oscillations in phantom perception

**Figure 4:**
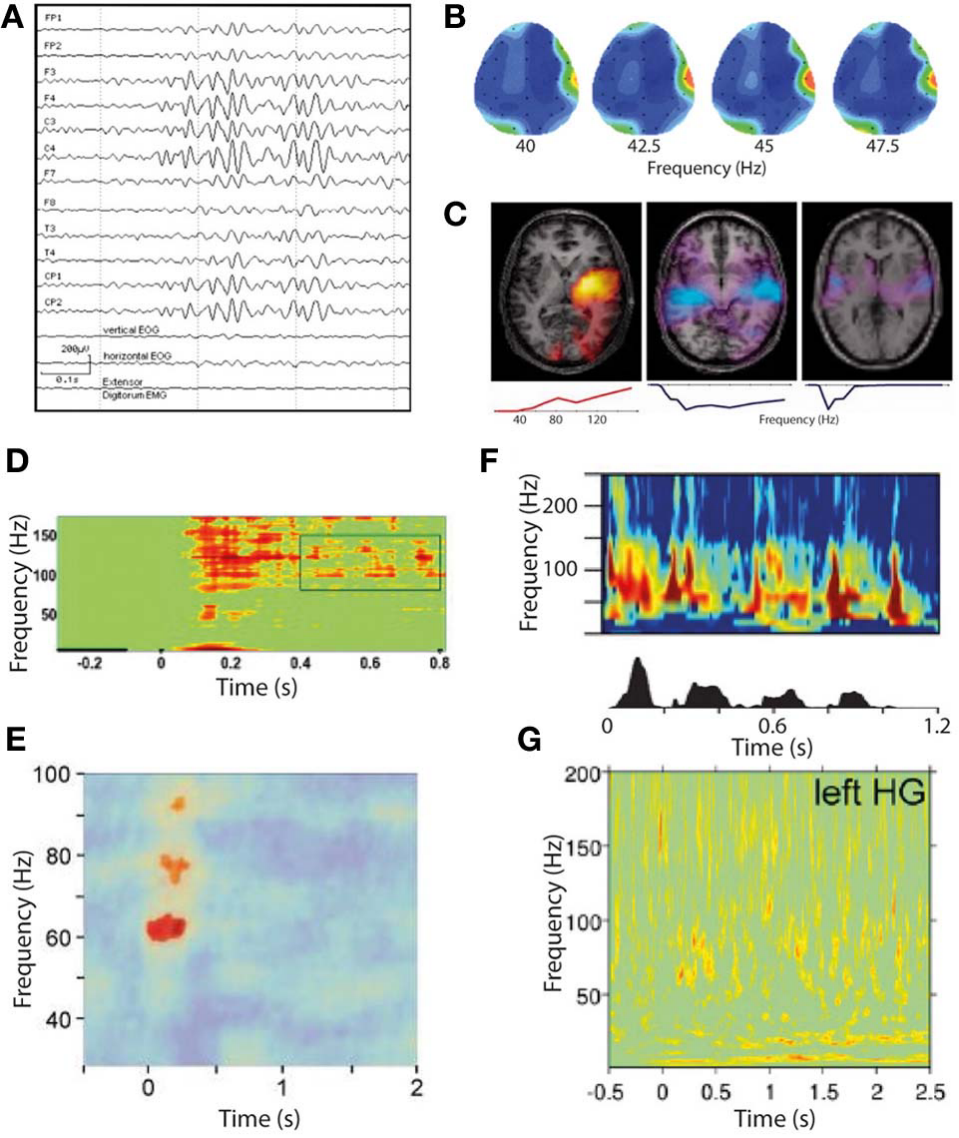
**Gamma oscillations in phantom and normal somatosensory and auditory perception. (A–C)** Gamma oscillations in phantom perceptual conditions are very high amplitude and easily visualized. **(A)** Raw scalp EEG waveforms from a patient with somatic phantom perception. Note the gamma oscillations dominate the entire EEG spectrum, whereas even the strongest gamma oscillations in response to external sensory stimuli cannot be seen without significant post-processing (reproduced with copyright-holder's permission from Baldeweg et al., 1998). **(B)** Scalp topography of resting-state gamma power in a patient with chronic tinnitus. Note the focus of gamma power, predominantly in a relatively narrow frequency band, over the right temporal lobe coinciding with auditory cortex (reproduced with permission from Ashton et al., 2007). **(C)** Individual patient correlates of increased tinnitus intensity (in response to small changes in perceived tinnitus intensity), in auditory cortex measured with MEG, occurring in a residual inhibition (RI; left) and residual excitation (RE; middle and right) paradigm. Brain slice overlays illustrate the spatial distribution of gamma power changes, and plots below illustrate the frequency spectra of these power changes. Note the positive correlation of gamma power with tinnitus intensity in RI and the negative correlation in RE. Also note the variable spectrum of the gamma oscillations, being broadband in two cases and narrowband in one (reproduced with permission from Sedley et al., 2012). **(D–G)** Gamma oscillations in response to external sensory stimulation are high-frequency, broadband and weak in amplitude. **(D)** Somatosensory cortex gamma oscillations in response to tactile stimulation, recorded with ECoG (reproduced with copyright-holder's permission from Ray et al., 2008). **(E)** Somatosensory cortex gamma oscillations in response to painful stimuli, recorded with MEG. Note the weaker and more limited response obtained with MEG than with ECoG as in **(D)** (reproduced with copyright-holder's permission from Gross et al., 2007). **(F)** Auditory cortex gamma oscillations recorded with depth electrodes (upper) in response to a speech stimulus (speech envelope shown in lower panel), with a clear burst of gamma oscillations following each speech sound after a short delay (reproduced with permission from Nourski et al., 2009). **(G)** Auditory cortex gamma oscillations recorded with MEG in response to equivalent speech paradigm as in **(F)**. Note the very weak and indistinct response (despite procedural optimization; reproduced with copyright-holder's permission from Millman et al., 2013).

An important discovery has been the demonstration, with direct recordings from the modality-specific thalami of patients with phantom perception, of persistent low-frequency spike bursts (Jeanmonod et al., 1996). These are believed to be a paradoxical result of de-afferentation and consequent hyperpolarization, leading to a spontaneous burst firing mode. This spiking has been linked with delta and theta band oscillations in the sensory cortex to which the bursting thalamic neurons project, leading to a model of phantom perception generation called *thalamocortical dysrhythmia* (Llinás et al., 1999, 2005). This model was proposed, based largely on early limited evidence of increased resting-state gamma oscillations in the magnetoencephalograms of a small number of people with tinnitus and chronic central neuropathic pain, along with the theory that this gamma is triggered by the low-frequency delta/theta activity. While the thalamus has been proposed as a key site in the genesis of tinnitus, a large body of work has found increased spontaneous activity as early as the dorsal cochlear nucleus in tinnitus, which could also represent the primary drive to tinnitus (Kaltenbach and Godfrey, 2008). In either case, there is strong evidence that tinnitus-related neural activity is initiated earlier in the auditory pathway than cortex, thus the cortex must receive this signal as an excessive and abnormal input, rather than being the primary site of tinnitus generation. Studies on tinnitus have found increased resting-state gamma oscillations in auditory cortex associated with the presence of tinnitus (vs. control groups; Ashton et al., 2007; Weisz et al., 2007). Figure 4B illustrates an example of a “hot spot” of spontaneous gamma oscillations in the EEG of a patient with chronic tinnitus. Where this has been searched for, increased resting-state delta/theta activity was also associated with tinnitus (Weisz et al., 2007), implying that gamma was a response to this low-frequency drive as opposed to a primary initiating event in tinnitus genesis. Other observations linking gamma to tinnitus, but not delineating its specific role, include a positive correlation between tinnitus loudness (rated on a subjective visual analog scale) and resting-state auditory cortex gamma oscillations measured with EEG (van der Loo et al., 2009). As there was no within-subject correlation of this relationship over time, we cannot infer a specific role of gamma in tinnitus from this study alone. Direct electrical stimulation of non-primary auditory cortex, in a single tinnitus patient, was found to induce elimination of tinnitus lasting beyond the end of stimulation which was accompanied by a reduction in both theta and gamma oscillations (De Ridder et al., 2011b). The same study found that after months of treatment, a persistent reduction in theta and gamma oscillations in auditory cortex detected with scalp EEG. Elimination of this theta-gamma neural signature (transiently and long-term) coinciding with the contemporaneous elimination of tinnitus is compelling evidence for a role of gamma oscillations in tinnitus, but again in each case gamma could simply be driven by theta oscillations regardless of the nature of its role. It has recently been found that “overcompensation” music therapy, a counterproductive experimental treatment that exacerbated tinnitus, was found to increase auditory cortex gamma oscillations following a period of treatment (Vanneste et al., 2013). Similarly, transient tinnitus occurring in amateur rock musicians experiencing high levels of noise exposure was found to be associated with increased gamma oscillations in auditory cortex (Ortmann et al., 2011). However, the magnitude and laterality of the gamma increases did not have a clear relationship to the laterality and magnitude of perceived tinnitus, with the authors highlighting that the gamma oscillations showed a closer relationship to transient hearing loss than transient tinnitus. Examining the converse situation, it has been found that successful treatment of tinnitus with a form of acoustically-delivered neuromodulation was associated with long-term reductions in auditory cortex gamma power as measured with EEG, but only as part of a general normalization of all spontaneous EEG correlates of tinnitus, including abnormalities in delta, theta, alpha and gamma bands (Tass et al., 2012). Despite this handful of studies linking gamma oscillations to tinnitus, some studies strongly expected to detect gamma changes in tinnitus have failed to do so. During tinnitus masking with an auditory stimulus, the perception of tinnitus was eliminated, accompanied by a reduction in low-frequency delta oscillations but no changes in gamma oscillations as measured with MEG (Adjamian et al., 2012). A study of MEG in residual inhibition (RI; suppression of tinnitus loudness lasting beyond the end of a masking stimulus) similarly found reduced delta band power but no detectable gamma changes despite specifically searching for them (Kahlbrock and Weisz, 2008). These studies were conducted at group level; a group-of-case-studies approach using MEG found that auditory cortex gamma suppression was associated with RI at an individual subject level in the majority of participants, as was auditory cortex delta/theta suppression (Sedley et al., 2012).

### Evidence for a “positive” role of gamma oscillations in phantom perception

The strongest evidence for a positive role of gamma in tinnitus, as discussed in section Non-Discriminatory Studies of Gamma Oscillations in Phantom Perception, is that resting-state auditory cortex gamma oscillations positively correlate with subjective tinnitus loudness (van der Loo et al., 2009). While this is compatible with a positive role for gamma in generating the tinnitus percept, very little information was given about what the subjective scale was actually rating. Theoretically there is an important distinction between *overall* tinnitus loudness, indicating how loud their tinnitus is on a typical day compared to a range of environmental sounds, and *current* tinnitus loudness, indicating how loud the tinnitus is on the day of study with respect to its usual range of fluctuation. *Overall* tinnitus loudness would likely include an increased cortical input, and therefore a positive association with gamma magnitude would not be very informative, whereas a tight association between *current* tinnitus loudness and gamma would be more compelling for a positive role of gamma, provided it were not accompanied by equivalent delta/theta changes. Unfortunately the study did not make this distinction, so a role of gamma cannot be clearly inferred from its findings. Outside of tinnitus, gamma oscillatory abnormalities have been reported in a highly unusual single case of idiopathic phantom somatosensory perception (Baldeweg et al., 1998). In this case, the patient experienced recurrent transitory somatic sensations, particularly in the oro-facio-cervical area, that were not clearly the result of de-afferentation, psychiatric or neurological illness. During periods of hallucination, scalp EEG clearly recorded gamma oscillations whose source localized to the somatotopically appropriate part of S1 to explain the hallucination. Figure 4A illustrates these gamma oscillations in the context of the raw EEG waveforms recorded from the patient.

### Evidence for a “negative” role of gamma oscillations in phantom perception

Examining the specific role of gamma oscillations in phantom perception should ideally involve dynamic modulations of the percept's intensity, along with a way of dissociating the gamma response from the low-frequency cortical inputs that trigger it. This is difficult to achieve, but has been fortuitously accomplished through a phenomenon called residual excitation (RE; Sedley et al., 2012). In this phenomenon, an auditory stimulus is presented (in this case band-passed noise lasting tens of seconds) and after the stimulus offset there is in increase in tinnitus loudness that lasts for seconds to tens of seconds. In all four tinnitus patients exhibiting the phenomenon, RE was accompanied by dramatic and typically bilateral reductions in auditory cortex gamma power, despite involving significant increases in perceived tinnitus intensity (Sedley et al., 2012). These gamma changes occurred in the absence of delta/theta changes in auditory cortex, and typically without any oscillatory power changes elsewhere in the brain, suggesting a primary effect of gamma oscillations in reducing tinnitus intensity. Figure 4 (**C**: middle and right panels) illustrates the isolated auditory cortex gamma power reductions correlating with residual excitation in two of these patients. Additionally, one patient's RI (reduction in tinnitus) was accompanied solely by an increase in auditory cortex gamma oscillations, again finding an inverse correlation between tinnitus intensity and gamma oscillation magnitude, in the absence of other oscillatory power changes.

### What type of gamma oscillation is associated with phantom perception?

As discussed in section Types of Gamma Oscillations in the context of normal perception, narrowband gamma (which is also unexpectedly high amplitude and persistent for the stimulus duration) appears only to occur in visual cortex, in response to very specific stimuli, while to our knowledge there have been no demonstrations of a similar mode of gamma in auditory or somatosensory cortices. However, there are some characteristics of the gamma oscillations associated with tinnitus, mentioned in sections Non-Discriminatory Studies of Gamma Oscillations in Phantom Perception, Evidence for a “Positive” Role of Gamma Oscillations in Phantom Perception and Evidence for a “Negative” Role of Gamma Oscillations in Phantom Perception, that invite the question of whether they might be related to narrowband visual gamma. The first of these is that, while gamma oscillatory responses to even very salient external auditory stimuli are extremely weak when recorded with MEG (Nourski et al., 2009; Sedley et al., 2012; Millman et al., 2013) and require large numbers of trials and optimized source reconstruction techniques to detect at all, gamma oscillations in response to comparatively quiet tinnitus are readily detectable using basic MEG montages (Weisz et al., 2007) and scalp EEG (Ashton et al., 2007; van der Loo et al., 2009), which detects auditory cortex signals less strongly than MEG due to the tangential orientation of core auditory cortex. Although not definitely comparable to tinnitus, the patient with idiopathic somatic hallucinations mentioned in Section Evidence for a “Positive” Role of Gamma Oscillations in Phantom Perception had gamma oscillations so strong that they dominated the raw scalp EEG sensor waveforms, a phenomenon that to our knowledge is unique to this case. Figure 4 illustrates examples of the unexpectedly strong gamma oscillations associated with phantom perception (**A–C**), which are unlike the comparatively weak somatosensory and auditory cortex gamma observed in response to sensory stimulation (**E** and **G**, respectively). For comparison, equivalent and relatively strong intracranially-recorded auditory and somatosensory gamma oscillations to those shown in e and g are shown (in **D** and **F**, respectively) to demonstrate how weakly these gamma oscillations are detected with non-invasive methods in healthy subjects. On top of its unexpectedly high amplitude, the frequency band of these gamma oscillations associated with phantom perception was often very narrow and comparatively low frequency, which is at odds with somatosensory gamma in general which is generally much weaker and in a higher, broader frequency range (Bauer et al., 2006; Gross et al., 2007). In tinnitus, gamma frequency bands show considerable variation; some studies have not presented the frequency spectrum but instead restricted analyses to narrow bands, e.g., 30–45 Hz (van der Loo et al., 2009; De Ridder et al., 2011b) and 55–60 Hz (Weisz et al., 2007), while at the individual subject level there appear to be both patients with narrowband gamma centered at around 40–60 Hz and those with broadband gamma extending to at least 150 Hz (Sedley et al., 2012). Figure 4 (**C**: lower row of panels) illustrates the gamma spectra associated with three of these tinnitus patients, including low-frequency, high-frequency and broadband spectra. Thus, it appears that tinnitus-related gamma oscillations are unusually high-amplitude, and are sometimes surprizingly low and/or narrowband in frequency. Why this is the case remains unknown. One must assume that either these oscillations represent a different neural process to the usual auditory stimulus-induced gamma (either similar to visual narrowband gamma, or a separate process altogether), or that they are the same process that has undergone a transformation toward low frequency and high amplitude on account of the persistence of the sensory object with which they are associated.

## Epilepsy

### Pathological “gamma” oscillations in epilepsy

High frequency oscillations (*ripples* and *fast ripples;* typically above 70–100 Hz) are strongly implicated in epileptogenesis, being associated with both interictal spikes (Andrade-Valença et al., 2012) and preceding epileptic seizures (Traub et al., 2001). However, there is evidence that they likely reflect a different neuronal process to physiological oscillations at the same frequencies (Bragin et al., 2004) therefore this review will not deal with these pathological oscillations. However, there are some studies that link physiological gamma oscillations with epilepsy, and these are discussed in the following section.

### Non-discriminatory studies of gamma oscillations in epilepsy

**Figure 5:**
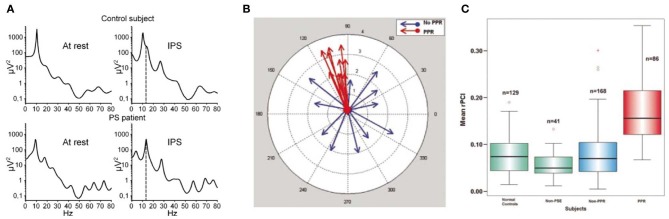
**Abnormalities of the gamma response to intermittent photic stimulation (IPS) in photosensitive epilepsy (PS). (A)** Logarithmic power spectra of local field potential in occipital EEG electrodes at rest (left) and during intermittent photic stimulation at 14 Hz (IPS; right), in a healthy control (upper) and a photosensitive epilepsy patient (lower). In the IPS condition, the stimulation frequency (14 Hz) is indicated by the dashed line. Note the resting-state power spectrum (left) shows more distinct peaks, mainly in the gamma range, in the PS patient (lower) than in the healthy control (upper); also that during IPS (right) the PS patient shows clear peaks at harmonics of the stimulation frequency, again mainly in the gamma range, indicating excessive entrainment of ongoing oscillatory brain rhythms to extrinsic stimulation in PS (reproduced with copyright-holder's permission from Visani et al., 2010). **(B)** Illustration of the concept of the Phase Clustering Index (PCI) in relation to IPS leading to (red) and not leading to (blue) a photoparoxysmal response (PPR; a type of stimulus-induced epileptiform activity). Each arrow represents one harmonic of the IPS frequency, with its direction indicating the phase of the oscillation. In the “no PPR” condition (blue) the oscillatory phases at the harmonic frequencies appear to be randomly distributed, while in the “PPR” condition (red) they are clustered around a common phase angle. The PPR is quantified as the vector sum of all the arrows in a particular condition. **(C)** Relative phase clustering index (rPCI) values, in response to IPS, in (left to right) normal controls, patients with non-photosensitive epilepsy, PS patients in trials not triggering a PPR, and PS patients in trials triggering a PPR. Note only trials associated with a PPR are associated with an abnormally high rPCI (reproduced with copyright-holder's permission from Parra et al., 2003).

It has been found that the particular stimulus properties giving rise to narrowband gamma in the visual system are the same ones that tend to trigger photoparoxysmal discharges (interictal epileptiform activity) in patients with photosensitive epilepsy (Adjamian et al., 2004). While this immediately raises the possibility that these gamma oscillations therefore have a role in epilepsy, it does not help to establish whether the gamma oscillations are a precursor to epileptogenesis, or whether they are triggered by epileptogenic stimuli and act to prevent a transition to epileptiform activity. Further evidence of a link between gamma and ictal activity comes from the study of children with Rolandic epilepsy (Doesburg et al., 2013). This found that children with the highest motor cortex gamma induced by median nerve stimulation, measured with MEG, showed the strongest gamma synchrony in motor cortex ictally, recorded with intracranial EEG. The study also found that clinical motor impairment was correlated with the magnitude of stimulus-induced gamma oscillations. Other intracranial EEG work has found that the period of unconsciousness following generalized seizures is accompanied by very large increases in broadband gamma power (Pockett and Holmes, 2009). Work in mouse hippocampal slices has found that, under epileptogenic conditions, periods of gamma oscillations alternate with epileptiform discharges, with transitions to the latter occurring when pyramidal cell to inhibitory interneuron synapses are sufficiently depressed (Traub et al., 2005). Still, however, these findings do not help determine whether the observed gamma oscillations were a precursor to epileptiform activity, or represent the final stages of a “brake” mechanism either just before the threshold to epileptogenesis is crossed or immediately following a de-escalation from epileptiform activity to physiological oscillations. Further to studying the relationship of gamma oscillation magnitude with epilepsy, abnormalities of the gamma spectrum have been studied. It has been found that the resting-state gamma spectrum in patients with photosensitive epilepsy contains more sharp peaks than in control subjects (Visani et al., 2010), as shown in Figure 5 (**A**: left panels). During intermittent photic stimulation (which is used to induce interictal epileptiform activity) the photosensitive epilepsy patients displayed more peaks in the gamma spectrum that were harmonics of the stimulation frequency, as illustrated in Figure 5 (**A**: right panels). A measure of neuronal oscillatory entrainment to the photic stimulation rhythm, the *phase clustering index* (PCI), has been proposed; in patients with photosensitive epilepsy, the PCI was much higher in photic stimulation trials that progressed to epileptiform activity (Parra et al., 2003). Figure 5 illustrates these abnormalities of the gamma spectrum (**A**), and PCI associated with photic stimulation (**B,C**). These findings are important in demonstrating that abnormalities of gamma synchrony are important in epileptogenesis. However, the findings can plausibly be interpreted as indicating a positive or negative role for gamma in this context; in a positive role, one could argue that the hyper-synchrony leads to increased summation of gamma power and crossing of a threshold into epileptogenesis, while a negative role explanation might suggest that the entrainment of gamma oscillations to external or erroneous rhythms prevents them from exerting their usual inhibitory control on local neuronal activity, leading to epileptogenesis through disinhibition. Put another way, peaks in the gamma spectrum, and excessive gamma phase locking to external stimulation, could reasonably be argued to either lead to excessively active gamma at specific times or frequencies, or to reflect inability of gamma oscillations to carry out their normal function due to being excessively clustered to specific times or frequencies.

## Conclusions

### Key findings

We have found that the most common factor limiting interpretation of studies on gamma oscillations is a definite or potential increase in input to the relevant cortical area as a function of the effect under study (see Figure 1). Where this is the case, gamma oscillation changes can always be plausibly explained as a secondary consequence of altered cortical input. Similarly, if gamma oscillation changes occur only as part of an exaggeration of the whole stimulus-induced response pattern then it is difficult to claim any special status of the gamma oscillations over and above any other changes in local neural activity. We have highlighted several clear distinctions between “broadband” and “narrowband” gamma, and have thus treated them as separate entities. Narrowband gamma, in the setting of normal perception, appears only to occur in visual cortex, and only in response to stimuli known to cause perceptual surround suppression (Tadin et al., 2003; Adjamian et al., 2004; Jia et al., 2011). This observation, along with others such as an inverse correlation with broadband gamma and multi-unit activity (Jia et al., 2011) makes a strong case for narrowband gamma oscillations serving an inhibitory role, which could include mediating surround suppression. We have also discussed evidence that narrowband gamma likely mediates perceptual suppression of unattended stimuli (Fries et al., 2002). However, against a simple inhibitory explanation, evidence suggests that narrowband visual gamma may have an active role in the forward-transmission of stimulus information (Gregoriou et al., 2009; Bosman et al., 2012; Jia et al., 2013; Roberts et al., 2013). There is also evidence that the magnitude of narrowband gamma oscillations correlates with the degree to which visual stimuli are consciously perceived even when all other stimulus features remain unchanged (Schurger et al., 2006; Wyart and Tallon-Baudry, 2008), suggesting a positive role in conscious sensory representation. Narrowband gamma seems to be related to epileptogenesis (Parra et al., 2003; Adjamian et al., 2004; Visani et al., 2010; Doesburg et al., 2013) but, while a role in suppressing epileptiform activity is plausible, there is a lack of direct evidence in this context to favor either an inhibitory or excitatory role. Broadband gamma seems to occur in various cortical areas, and unlike narrowband visual gamma it is positively coupled to other measures of mass neural activity (Mukamel et al., 2005; Canolty et al., 2006). The role of broadband gamma seems to be largely positive, being associated with attention (Bauer et al., 2006), gestalt perception (Lachaux et al., 2005), object recognition (Fisch et al., 2009), memory performance (Osipova et al., 2006) and perceptual salience (Griffiths et al., 2010). However, several key findings are incompatible with gamma merely being coupled to perceptual salience, including not persisting for the whole stimulus duration (Griffiths et al., 2010; Sedley et al., 2012), occurring equally in response to missing stimuli as to present ones (Fujioka et al., 2009) and resulting preferentially from incongruent stimuli rather than salient ones (Arnal et al., 2011). Conversely very little evidence, from animal studies, supports an inhibitory role of broadband gamma (Macleod et al., 1998; Weinberger et al., 2006), which if present might increase stimulus specificity by inhibiting inappropriate downstream neural responses. Interpretation of the role of broadband gamma is made more difficult by fluctuations in the local field potential in this frequency range potentially representing varying combinations of multi-unit spiking and true oscillations (Scheffer-Teixeira et al., 2013). Unifying explanations as to the role of broadband gamma include it being simply a signature of neural activation (Merker, 2013), and generating prediction errors in a predictive coding model of brain function (Arnal and Giraud, 2012), but neither of these is close to proven. Gamma oscillations are clearly associated with the presence of phantom perception, but their characteristics (including very high magnitude and atypical frequency bands) mean it is not clear whether they represent the same process as narrowband or broadband perceptual gamma, or a different process. Most evidence for the role of these oscillations is non-discriminatory, but a single study on dynamic correlates in individual patients strongly favors an inhibitory role (Sedley et al., 2012).

### Outstanding questions

While there is clear and increasing evidence for both specific positive and inhibitory roles of narrowband gamma in normal perception, its role with respect to epileptogenesis remains very unclear, and direct experimental evidence of its effect on epileptiform activity is required in order to address this question. Narrowband gamma has only been convincingly demonstrated in visual cortex, and it is unclear whether it is biologically restricted to the visual system, or rather that it can exist in other sensory modalities and the appropriate stimuli have simply not been tested. Also unclear is to what extent gamma in phantom perception relates to narrowband gamma. If it is the same process then it remains unknown why phantom perception in auditory and somatosensory cortices can generate it but external stimulation to the same cortices apparently cannot, while if it represents a different process to sensory gamma oscillations then questions remain about what this process is and why it should be unique to phantom perception.

### Recommendations for future research

We suggest that future research should aim to selectively focus on the role of gamma oscillations by: accounting for the strength of both cortical input and onward connections, e.g., by experimental design or quantitative estimation; ideally using a model of effective connectivity (Chen et al., 2008) to explicitly model the effect of gamma on other neural activity; accounting for all levels of the cortical hierarchy where more than one level is interacting. As there appear to be multiple fundamental differences between them, studies should make it explicit whether they are commenting on narrowband or broadband gamma, and a standard nomenclature should be adopted to distinguish the two. Additionally, efforts should be made, in the case of broadband gamma, to differentiate spectral leakage of multi-unit activity from true oscillations. Present evidence suggests that this may be achievable by examining frequency band and relationship between “gamma” power and theta phase, but as this is an emerging issue it is likely that additional methods of distinction will be available in future. As well as following the suggestions above, research on photosensitive epilepsy should aim to directly relate, on an individual trial basis, stimulus-induced gamma oscillations to epileptiform activity induced by the same stimuli. Research on gamma in phantom perception would be greatly enhanced by establishing whether the gamma associated with them is a variant of the narrowband or broadband gamma associated with normal perception. This goal would be aided by trying to establish if there are conditions in which externally-presented stimuli can be made to induce gamma responses resembling those found in phantom perception. Finally we emphasize to researchers studying them that although it is attractive to assume that gamma oscillations are a positive process that holds the answers to the brain's highest order functions, there is far from sufficient or unilateral evidence to support this assertion; thus interpretation of each study's findings should be approached without a default prior hypothesis of gamma serving a positive perceptual role.

### Conflict of interest statement

The authors declare that the research was conducted in the absence of any commercial or financial relationships that could be construed as a potential conflict of interest.
